# The Two Faces of Wheat

**DOI:** 10.3389/fnut.2020.517313

**Published:** 2020-10-21

**Authors:** Herbert Wieser, Peter Koehler, Katharina A. Scherf

**Affiliations:** ^1^Retired, Freising, Germany; ^2^Biotask AG, Esslingen am Neckar, Germany; ^3^Department of Bioactive and Functional Food Chemistry, Institute of Applied Biosciences, Karlsruhe Institute of Technology (KIT), Karlsruhe, Germany

**Keywords:** allergy, baking, breeding, celiac disease, gluten, non-celiac gluten sensitivity (NCGS), nutritional value, wheat

## Abstract

Wheat-based foods have been staple foods since about 10,000 years and constitute a major source of energy, dietary fiber, and micronutrients for the world population. The role of wheat in our diet, however, has recently been scrutinized by pseudoscientific books and media reports promoting the overall impression that wheat consumption makes people sick, stupid, fat, and addicted. Consequently, numerous consumers in Western countries have started to question their dietary habits related to wheat consumption and voluntarily decided to adopt a wheat-free diet without a medical diagnosis of any wheat-related disorder (WRD), such as celiac disease, wheat allergy, or non-celiac gluten sensitivity. The aim of this review is to achieve an objective judgment of the positive aspects of wheat consumption as well as adverse effects for individuals suffering from WRDs. The first part presents wheat constituents and their positive nutritional value, in particular, the consumption of products from whole-grain flours. The second part is focused on WRDs that affect predisposed individuals and can be treated with a gluten-free or -reduced diet. Based on all available scientific knowledge, wheat consumption is safe and healthy for the vast majority of people. There is no scientific evidence to support that the general population would benefit from a wheat-free diet.

## Introduction

Wheat is one of the major crops grown worldwide with a production of 7.34 × 10^8^ tons on an area of 2.14 × 10^6^ km^2^, which is about the size of Greenland (1). Wheat-based foods have been staple foods since wheat was domesticated about 10,000 years ago, and they constitute a major source of macro- and micronutrients and energy (15–20% of the required intake) for the world population, especially in developing countries (2, 3). Many health benefits such as favorable weight management and reductions in the risks for cardiovascular diseases and type 2 diabetes have been shown to be associated with the consumption of wheat-based foods, especially made of whole grains (4–6). Moreover, many non-food products for daily use contain wheat constituents as valuable ingredients. As a result, the positive aspects of wheat were commonly unquestioned. On the other hand, wheat-based foods are known to cause wheat-related disorders (WRDs), such as celiac disease (CD), wheat allergy (WA), and non-celiac gluten sensitivity (NCGS) in predisposed individuals (7). In the last decade, wheat received an increasingly negative image due to a number of pseudoscientific books and press reports, which recommended the avoidance of wheat consumption for the general population, not only for those suffering from WRDs. As a consequence, increasing numbers of individuals in Western countries decided to adopt a gluten-/wheat-free diet even without clear indications of WRDs or medical advice. The percentages of individuals self-reporting a WRD among the general population were 15% in Australia (8), 13% in the United Kingdom (9), 10% in Brazil (10), 8% in Mexico (11), 6% in the Netherlands (12), and 3% in El Salvador (13). In light of this controversial debate, the aims of this review are to provide an objective judgment of the positive aspects of wheat consumption as well as adverse effects for individuals suffering from WRDs.

## Origins and Importance of Wheat

Wheat plants are grasses belonging to the monocot family Poaceae. Cultivated wheat (*Triticum* spp.) consists of three species: diploid (genome A^m^A^m^) einkorn (*T. monococcum* ssp. *monococcum*), tetraploid (AABB) emmer (*T. turgidum* ssp. *dicoccum*) and durum wheat (*T. turgidum* ssp. *durum*), and hexaploid (AABBDD) common wheat (*T. aestivum* ssp. *aestivum*) and spelt (*T. aestivum* ssp. *spelta*) (14). Using recent advances in sequencing techniques, the International Wheat Genome Sequencing Consortium (IWGSC) recently published a detailed description of the total genome of common wheat (cultivar Chinese Spring) and enabled access to 107,891 gene sequences (15). Einkorn developed from wild einkorn 1 (*T. monococcum* ssp. *boeticum*) and the cultivation of einkorn started around 10,000 years ago in the Fertile Crescent. The hybridization of a different wild einkorn (*T. monococcum* ssp. *urartu*) with an *Aegilops speltoides-*related species (BB) resulted in wild emmer (*T. turgidum* ssp. *dicoccoides*), the ancestor for domesticated emmer. Some subspecies of wild emmer developed free-threshing (naked) grains, known as durum wheat. When durum wheat crossed with *Aegilops tauschii* (DD), naked hexaploid common wheat evolved. Spelt most likely emerged from hybridization between *T. aestivum* and *T. dicoccum* (16).

Common (bread) wheat makes up about 95% of all wheat cultivated globally, and most of the remaining 5% is durum (pasta) wheat. Despite its ability to grow in variable environmental conditions, the selection of qualified sites and soils for wheat cultivation as well as suitable varieties and optimal crop management are important factors to ensure high yields (17, 18). With an estimated additional two billion people on the planet by 2050, food production needs to be increased despite challenges arising from climate change (19, 20). Improved wheat plants, resistant to frost, heat, drought, and/or salty soils, may increase the grain yield and the area suitable for wheat production and thus help ensure food security.

Wheat grains are dry one-seeded fruits (caryopses), in which fruit and seed coats are tightly linked. The husk is fused to the fruit coat in the hulled species einkorn, emmer, and spelt, which means that the husk cannot be separated from the grain by threshing. The grains consist of five main compartments with different constituents and biological functions: Fruit coat (pericarp) (4–5% of grain weight) and seed coat (testa) (≈1%) are the outer layers and surround the whole grain. The inner tissues (endosperm) comprise the aleurone layer (6–9%) and the starchy endosperm (80–85%). The germ (3%) located at the dorsal side of the caryopsis is the embryo, which includes a storage cotyledon and the embryonic axis. Dry (moisture content <12.5%), cool (<10°C), and pest-free storage of wheat grains protects against crop failure by providing a buffer to ensure nutrition security worldwide.

## The Smiling Face: Wheat Constituents and Their Health Benefits

The chemical composition of mature grains (water content ≈13%) varies in a relatively small range, although it is influenced by species, variety, and growing conditions. Carbohydrates, mainly present as starch (≈58%) and non-starch polysaccharides (NSP) (≈13%), are predominant, followed by proteins (≈11%), lipids (≈2%), and minerals (≈2%) (Table 1). Vitamins and phytochemicals occur in very small amounts (<0.1%) but are important due to their contribution to human health.

**Table 1:** Average contents of grain constituents of common wheat (21).

**Main constituents**	**(g/100 g)**	**Minerals**	**(mg/100 g)**	**Vitamins**	**(μg/100 g)**
Carbohydrates	73.2	Potassium	380	B_3_	5,100
Starch	58.2	Phosphorus	342	E	1,400
Non-starch polysaccharides	13.3	Magnesium	97	B_5_	1,200
Mono-, di-, oligosaccharides	1.7	Calcium	33	B_1_	455
Water	12.7	Sodium	7.7	B_6_	269
Protein	10.6	Iron	3.2	B_2_	94
Lipids	1.8	Manganese	3.1	B_9_	87
Minerals	1.7	Zinc	2.6	B_7_	6

### Mono-, Di-, and Oligosaccharides

Wheat grains contain only minor amounts (<0.1%) of the monosaccharides D-glucose and D-fructose, but about 0.5–1.6% of sucrose and 0.1–0.2% of maltose as disaccharides and 0.2–0.7% of the trisaccharide raffinose. The predominant oligosaccharides of wheat are 0.8–1.9% of fructans (22). Wheat fructans are of the graminan-type and comprise three or more fructose monomers linked via β-(2→1) and β-(2→6) glycosidic bonds and may also contain one glucose monomer. The degree of polymerization is 5–7 on average, but may also be below 5 or up to 17–19. Fructans are enriched in the bran and adhering endosperm (3–4%), but also present in the germ (≈2%) and endosperm (1–2%) (23). As part of fermentable oligo-, di-, and monosaccharides and polyols (FODMAPs), fructans and raffinose are metabolized by gut microbiota in the colon and may exert positive health effects similar to NSPs (see the Non-starch Polysaccharides section) by acting as prebiotics (24). In addition to their stimulatory effect on the composition and/or activity of beneficial gut bacteria, inulin-type fructans were shown to have direct immunomodulating and antioxidant protective effects on the gut mucosa (25, 26), but it is still unclear whether graminan-type fructans have the same effects as inulin-type fructans because studies with isolated cereal fructans are still needed (27). However, FODMAPs are also associated with intestinal complaints, as discussed below.

### Starch

Starch is restricted to the endosperm, where it is present as large lenticular granules (A-starch) and small spherical granules (B-starch). The major carbohydrates of both granule types are the polysaccharides amylose and amylopectin in a mass ratio of about 25%/75%. Amylose consists of mainly linear α-(1→4)-linked D-glucopyranose units that form a helical structure and has molecular masses between 80,000 and 1 million. Apart from α-(1→4) glycosidic bonds, amylopectin has a branched structure with α-(1→6) bonds occurring every 24–30 glucose units and reaches molecular masses as high as 100 million (28). Starch is important for end-product quality and important for human nutrition. It constitutes the main source of energy (11.4 kJ/g of wholegrain wheat flour and 13.1 kJ/g of white wheat flour) because starch is readily degraded by amylases during human gastrointestinal digestion to its glucose monomers.

Resistant starch is the proportion of starch that escapes digestion in the upper gastrointestinal tract and small intestine (29), resulting in a caloric value of 8 kJ/g compared to 15 kJ/g for rapidly digestible starch. It is inaccessible to amylases depending on the size, shape, and crystallinity of the starch granule, the complexation of amylose with lipids, proteins, and phosphate as well as food processing (30, 31). Similar to NSP, resistant starch forms part of dietary fiber (DF) because it is fermented by microbiota in the colon to short-chain fatty acids. Especially butyrate promotes normal colon function by serving as a source of energy for the epithelial cells and by lowering luminal pH, which, in turn, facilitates the growth, and proliferation of beneficial gut microbiota (32, 33). Further, health benefits of resistant starch include improved insulin sensitivity (34), reduced oxidative stress in the colon (35), and lower body fat levels (36). Despite the different effects discussed before, the EU register on Nutrition and Health Claims contains only one authorized entry asserting the reduction of post-prandial glycemic responses for foods where resistant starch is at least 14% of total starch. Wheat whole grain flour contains about 3% of resistant starch and thus contributes to the estimated intake of 3–6 g/day in Europe (37). However, this is still far from the recommended intake of 20 g of resistant starch/day that is needed to achieve the positive health effects (38).

### Non-starch Polysaccharides

NSPs include the cell wall polysaccharides arabinoxylans (AX, 5.5–7.4%), cellulose (1.7–3.0%), and β-glucans (0.5–1.0%) (22), but also minor contents of glucomannan, callose, xyloglucan, and pectins. Compared to amylose and amylopectin, NSPs are non-granular and belong to DF because human gastrointestinal enzymes are not able to cleave the predominant β-glycosidic linkages of the monosaccharide units. Depending on the wheat grain tissue, the composition of NSP varies between the endosperm, the bran, the aleurone, and the outer pericarp (39).

AX consist of a chain of β-(1→4)-linked D-xylopyranose residues carrying substitutions via (2→1)- and/or (3→1)-glycosidic bonds to α-L-arabinofuranose residues. Some arabinofuranose residues may additionally be linked to ferulic acid at C_5_, so that two adjacent AX chains may become crosslinked via diferulate following oxidation. The extent of diferulate crosslinking affects the solubility and viscosity of AX that can be subdivided into water-extractable (WE)-AX and water-unextractable (WU)-AX. Cellulose consists of linear β-(1→4)-linked D-glucopyranose units, whereas β-glucans are β-D-glucopyranose units linked via (1→3)- and (1→4)-glycosidic bonds. Compared to oats and barley, wheat β-glucans are poorly soluble. Wheat bran composition is significantly influenced by genotype × environment interactions, and the content of neutral detergent fiber ranged from 19 to 31%, that of acid detergent fiber from 5 to 10%, that of cellulose from 3 to 9% and that of hemicellulose from 14 to 21% (40).

All NSPs as well as lignin and fructans are summarized as DF. Wholegrain wheat flour contains 10.3–15.5% of total DF, whereas white flour only has 1.9–6.3% (41). Countless studies support the beneficial effects of wheat NSP on human health, including the comprehensive review by the UK Scientific Advisory Committee on Nutrition (SACN) (42). The most important effects include the regulation of colonic functions, protection against colonic cancer, normalization of serum lipid levels, and attenuation of post-prandial glucose response. A number of studies have reported the protective effects of wheat DF against colon, small intestinal, pancreatic, prostate, and breast cancer, with the effects on colorectal cancer being most evident (43–45). The influence of DF on the incidence of cardiovascular diseases has been the subject of many studies, and the relation between whole-grain intake and improved cardiovascular functions were clearly demonstrated with reduced risks for coronary events, stroke, elevated blood pressure, and hypertension (46). Many studies demonstrated that DF, enriched in whole grain products, has the ability to reduce insulin and glucose response significantly and, thus, reduce the occurrence of type 2 diabetes (47–49). Whether the consumption of DF-rich foods reduces appetite and contributes to weight control needs further clinical investigations (50). Currently, the EU Register on Nutrition and Health Claims contains two authorized entries for wheat bran fiber, one claiming reduced intestinal transit time and the other increased fecal bulk. Wheat AX is listed as suitable for the reduction of post-prandial glycemic responses. About 40 more entries related to DF were submitted, but not authorized, mostly due to the fact that the food constituent was not sufficiently characterized in relation to the claimed effects and a missing cause and effect relationship.

### Proteins

Together with yield, grain protein content is of primary importance for wheat breeding because the content and composition of wheat proteins largely determine the bread making quality of wheat (51). Both protein content and composition are influenced by genetic and environmental factors, but the interaction between the two complicates the identification of molecular markers linked to these traits (15). Among environmental factors, nitrogen fertilization is the most prominent determinant linked to protein content and composition, but other factors such as soil fertility, precipitation, temperature, and altitude also play a role (52). The protein content of wheat may range from 7 to 22%, but mostly lies between 10 and 15% (53). The highest percentages of proteins within the grain are found in the germ (34%), followed by the aleurone (23%) and 5–6% in the outer layers. Consequently, the protein content of whole-grain flour is usually about 2% higher compared to white flour.

The protein fraction of wheat consists of over 100 individual proteins, which can be classified according to their functions: storage proteins, metabolic proteins, protective proteins, and miscellaneous proteins with further specific functions. Gluten proteins are storage proteins located in the endosperm (54), representing around 80% of total grain protein and can be grouped into monomeric gliadins soluble in aqueous alcohols and insoluble polymeric glutenins (55, 56). Gliadins have molecular masses between 30,000 and 55,000 and are structurally differentiated into four types: ω5-, ω1,2-, and α- and γ-gliadins. Glutenins are linked by interchain disulfide bonds and have molecular masses between 600,000 and more than 10 million. The respective monomers are classified into low-molecular-weight (LMW) and high-molecular-weight (HMW) glutenin subunits (GS) with molecular masses around 30,000 and 75,000, respectively. Metabolic proteins include enzymes like hydrolases, which cleave starch (amylases), proteins (peptidases), and lipids (lipases), as well as other oxidoreductases, transferases, and further enzymes (57). The majority of protective proteins are enzyme inhibitors that inhibit external amylase and peptidase activities, some of which are bifunctional like α-amylase/trypsin inhibitors (ATIs). The group of miscellaneous proteins includes, e.g., puroindolines, purothionins, and agglutinins (58, 59).

The nutritional value of wheat proteins is determined by their relative contents of the essential amino acids valine, leucine, isoleucine, phenylalanine/tyrosine, tryptophan, threonine, methionine/cysteine, lysine, and the semi-essential arginine and histidine (60). Lysine is the first limiting amino acid in wheat grains, whereas the other essential amino acids are present in adequate amounts (41). The biological value of white wheat flour is estimated to be 52 and that of whole-grain wheat flour is 17–26% higher (61). This difference is due to the fact that white flour contains higher proportions of gluten proteins compared to whole-grain flour, and the amino acid composition of gluten is characterized by exceptionally high contents of non-essential glutamine (26–53%) and proline (10–29%) (62).

### Lipids

Wheat lipids constitute about 2–2.5% of the flour and can be classified into non-polar lipids (acylglycerols and free fatty acids) and polar lipids (phospholipids and glycolipids). The major components of non-polar lipids are triacylglycerols (≈40% of lipids) and free fatty acids (≈15%), while the percentages of mono- and diacylglycerols (≈1 and 4%, respectively) and sterol lipids (<1%) are low. Wheat phospholipids are composed of lysophosphatidylcholine (≈2%), phosphatidylcholine (≈1%), and <1% each of phosphatidylethanolamine, *N*-acylphosphatidylethanolamine, phosphatidylglycerol, and phosphatidyl inositol. Wheat glycolipids are comprised of digalactosyldiacylglycerol (DGDG) (≈15%), monogalactosyldiacylglycerol (MGDG) (≈4%), as well as DGMG and MGMG (about 1% each) (63, 64). Regarding nutritional benefits, wheat has high amounts of oleic acid (≈14%) as well as linoleic acid (≈60%) and linolenic acid (≈4%) as unsaturated fatty acids, and therefore a favorable ratio of unsaturated to saturated fatty acids of about 78%/22% (21). Wheat also contains phenolic lipids, known as alkylresorcinols (1,3-dihydroxybenzene derivatives with an odd-numbered alkyl chain at position 5 of the benzene ring) that may serve as markers of whole-grain cereals in food (65) and as biomarkers of whole-grain wheat intake (66). They have been reported to prevent colon cancer in mouse and *in vitro* models based on their antimutagenic and apoptotic activity (44, 45).

### Vitamins and Minerals

Wheat grains are important sources of vitamin E (mainly α-tocopherol) and B vitamins, especially thiamine (B_1_), riboflavin (B_2_), niacin (B_3_), pantothenic acid (B_5_), pyridoxine (B_6_), and folates (B_9_) (Table 1). Whole-grain flours have considerably higher vitamin contents than white flours because vitamins are predominantly found in the bran and germ. One important point to consider related to nutrition is the bioavailability of vitamins. For example, most of the niacin present in wheat bran is bound, and only about 10–20% was found to be bioavailable (67).

The major minerals are potassium, phosphorus, magnesium, and calcium, followed by zinc, manganese, and iron in lower amounts (Table 1). Copper and selenium are trace minerals. All vitamins and minerals present in wheat have well-known functions in supporting growth and in maintaining the health and well-being of humans.

### Phytochemicals

Wheat grains contain small amounts of phytochemicals that are defined as non-nutritive biologically active molecules that function in the human body to achieve health benefits, promote well-being, and prevent certain disease processes. The two major classes of phytochemicals found in wheat are phenolic compounds and terpenoids, derived from the shikimate and mevalonate or methylerythritol phosphate biosynthetic pathways, respectively (22, 68). Phenolic compounds are a structurally diverse group comprising phenolic acids (e.g., cinnamic acid and benzoic acid derivatives), flavonoids (e.g., flavanols and anthocyanins/anthocyanidins), and lignans. Terpenoids include sterols and stanols, with β-sitosterol as the primary compound in wheat, that may occur either as free form, esterified, glycosylated, or acylated and glycosylated. Other terpenoids are tocopherols and tocotrienols (α-tocopherol is commonly known as vitamin E) as well as carotenoids that can be subdivided into oxygen-containing xanthophylls (e.g., lutein and zeaxanthin) and oxygen-free carotenes (e.g., α- and β-carotene). Depending on their molecular structures, some carotenes may be converted to vitamin A in humans. With phytochemicals being mainly located in the aleurone and bran, they occur in the mg/kg range in whole-grain flours, but with a wide range of concentrations determined by natural genetic and environmental variations (69, 70).

Phenolic acids are known as strong antioxidants (71, 72), and there is evidence that phenolic compounds improve vascular functions in humans (73) and may have antitumor activity (44, 74). Considering the low overall concentrations of phytochemicals in whole-grain flours and absorbance rates of 5–10% in the human small intestine, direct antioxidant effects appear to be unlikely. However, the remaining 90–95% of phytochemicals are transferred to the colon where they are metabolized by microbiota and may exert positive effects on colon health through this route.

### Wheat Constituents Related to Consumption

**Figure 1:**
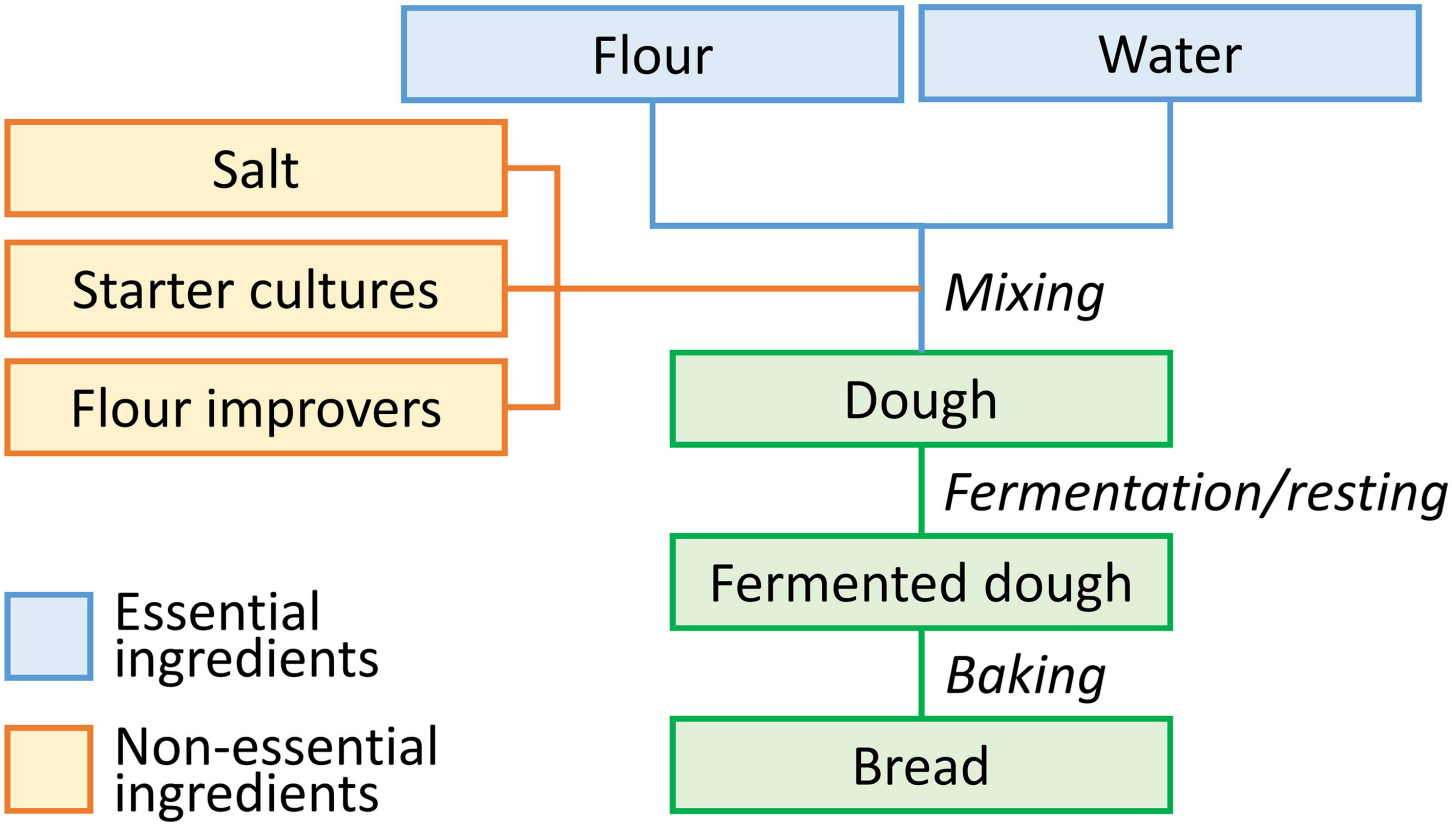
Schematic representation of the bread-making process.

In summary, wheat grains contain low amounts of sugars, sodium, fat, saturated fatty acids, and are free of cholesterol, all of them considered to restrict health. As most components related to health are concentrated in the outer layers (bran) and germ of the grain, their contents are reduced in flours with low extraction rates (e.g., white flour), which are used to make the majority of wheat products (e.g., bread, pasta, and noodles) consumed in Western countries (Figure 1). Products made from white flour are usually preferred by consumers because the high bran content of wholegrain flour may give a dark color, bitterness, gritty texture, and short shelf life. Changing consumer preferences is difficult, and thus, whole-grain intake is still below daily recommendations in most countries. To improve the situation, research and development should be focused on combining innovative processing with better product quality to increase the utilization of whole-grain flours. Moreover, physicians, nutritionists, and the media should advocate the consumption of products made from whole grains (75).

Altogether, the consumption of wheat-based foods, especially whole-grain products, is highly recommended owing to excellent nutritional profiles and their importance as sources of energy, proteins, DF, B, and E vitamins, minerals, and different micronutrients, all contributors to a healthy diet. Depending on the average annual per capita consumption, wheat contributes different amounts of nutrients in relation to the overall diet. The average per capita consumption of wheat worldwide was 67.0 kg (2016–2018), but with considerable regional variations ranging from 50.4 kg in Africa to 109 kg in Europe, considering continents, but even ranging from 16.2 kg in Thailand to 209.7 kg in Turkey, considering countries (76). In Germany, wheat consumption contributes 23% of energy, 34% of digestible carbohydrates, 34% of protein, 24% of DF, and 20–30% of vitamins and 10–20% of minerals compared to the recommended average intake values (77). However, considering the wide span of per capita wheat consumption, these values will vary in the same wide range and the contribution of wheat to dietary nutrient intake needs to be considered individually for each country.

## The Sad Face: Wheat-Related Disorders

**Figure 2:**
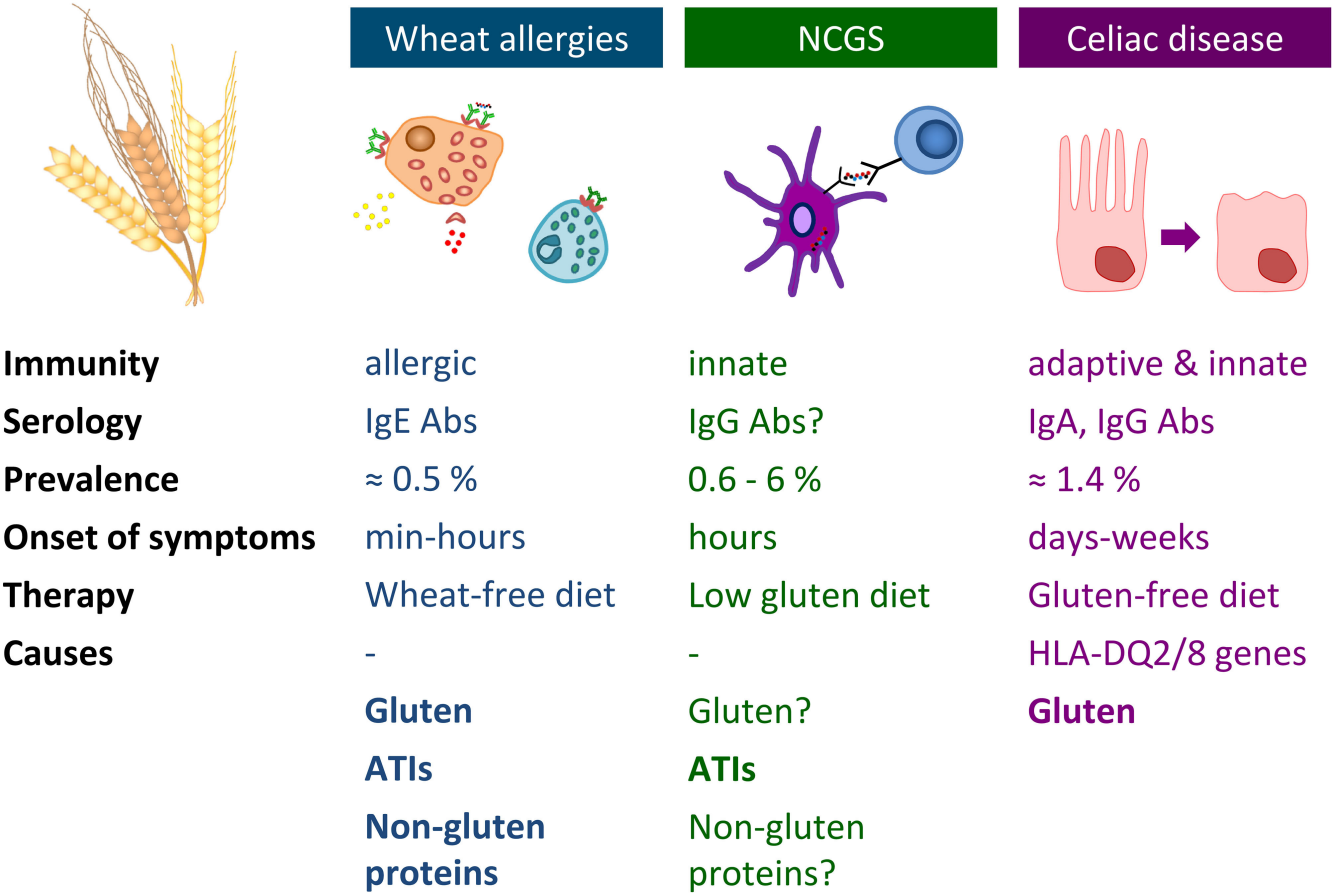
Overview of wheat allergy, non-celiac gluten sensitivity, and celiac disease. Abs, antibodies; ATIs, α-amylase/trypsin-inhibitors; HLA, human leukocyte antigen; Ig, immunoglobulin; NCGS, non-celiac gluten sensitivity.

Immune-mediated adverse reactions to wheat may occur in predisposed individuals. These hypersensitivities commonly referred to as wheat-related disorders (WRDs) can be classified into CD, gluten ataxia, and dermatitis herpetiformis characterized by an autoimmunogenic response (IgA and IgG antibodies), into IgE- and non-IgE-mediated WA and into NCGS characterized by an innate immune response (78) (Figure 2). A subgroup of patients with diarrhea-predominant irritable bowel syndrome may also be affected by wheat consumption.

### Celiac Disease

#### Definition and Prevalence

CD belongs to the most common food-related lifelong disorders worldwide. It is defined as a chronic immune-mediated small intestinal enteropathy precipitated by exposure to dietary gluten in genetically predisposed individuals (7). The term “gluten” comprises the closely related storage proteins of wheat (gliadins and glutenins), rye (secalins), and barley (hordeins). No species or variety of these cereals is currently safe for patients with CD. Recent epidemiological data suggest a mean worldwide prevalence of 1.4% [1.1–1.7%] based on positive serology and of 0.7% [0.5–0.9%] based on biopsy-confirmed diagnosis (79). The serology-confirmed prevalence values were 1.1% in Africa, 1.3% in Europe and South America, 1.4% in North America and Oceania, and 1.8% in Asia. Only people from sub-Saharan Africa appear to be hardly affected. There are considerable regional differences in seroprevalence ranging as high as 2.1–8.5% in Algeria, Czech Republic, India, Israel, Mexico, Malaysia, Saudi Arabia, Sweden, Portugal, and Turkey, or as low as 0.2–0.8% in Estonia, Germany, Iceland, Libya, Poland, Spain, and Switzerland. CD can occur at any age, but the biopsy-confirmed prevalence was significantly greater in children (0.9%) than in adults (0.5%) and also higher in female (0.6%) compared to male (0.4%) individuals. The worldwide prevalence of CD increased from 0.6% [0.5–0.7%] (years 1991–2000) to 0.8% [0.5–1.0%] (years 2001–2016), but the reasons are still unknown, although environmental factors are most likely (80).

#### Causes

A combination of environmental and genetic factors is necessary to trigger CD in susceptible individuals. Consumption of gluten from wheat, rye, or barley is the decisive environmental factor necessary for CD onset. Although comparative data on the CD activity of single gluten proteins is unavailable, most findings suggest that all gluten proteins are relevant. The unique structural features of gluten proteins are long repetitive amino acid sequences rich in glutamine and proline (62). These sequences are resistant to human gastrointestinal digestive enzymes, so that high amounts of long gluten peptides reach the upper small intestinal mucosa, pass the epithelium, and arrive at the lamina propria, where CD-specific immune reactions are induced (81). Recent evidence suggests that non-gluten proteins, such as ATIs, may also be involved in fueling the CD-specific immune response (82, 83).

The genetic susceptibility to develop CD is associated with the major histocompatibility class II genes on chromosome 6 coding for human leukocyte antigens (HLA)-DQ2 and -DQ8. HLA-DQ proteins are heterodimeric receptors expressed on the surface of antigen-presenting cells (APCs) that are responsible for binding gluten peptides and presenting them to gluten-specific CD4^+^ T cells. Depending on genetic expression patterns, either the HLA-DQ2.5, -DQ8, or -DQ2.2 heterodimers are present and associated with a very high, high, or low predisposition for CD, respectively (84, 85). These different levels of CD risk are due to HLA-DQ2.5 being capable of binding a large repertoire of gluten peptides that are resistant to gastrointestinal degradation, whereas HLA-DQ8 and -DQ2.2 bind a small to very small selection of gluten peptides that are also less resistant to degradation (86). About 95–97% of CD patients are HLA-DQ2/8 positive, but this genetic predisposition is also present in about 30% of the healthy population. Thus, the absence of HLA-DQ2/8 is a reliable criterion to exclude CD, but its presence is not sufficient to cause CD. A variety of non-HLA genes, mostly encoding for T cells or APCs, has been associated with CD development, but each of these genes most likely only contributes a small percentage to increase the risk of CD (87). It is interesting to note that there is a significant correlation between the level of wheat consumption, the frequency of HLA-DQ2/8 and the prevalence of CD, but with several outlier populations in regions such as northwestern India, northern Africa, Mexico, Finland, and Russia. For example, the prevalence of CD in Algeria is among the highest worldwide (5.6%), whereas that of Tunisia is very low (0.3%), although both countries share similar levels of wheat and barley consumption and frequencies of HLA-DQ2/8 (84). This discrepancy can only be explained by further environmental factors that cause a loss of tolerance to dietary gluten and initiate CD. The most likely factors are infections (rotavirus, adenovirus 12), changes of intestinal microbiota, increased small intestinal permeability, and the so-called hygiene hypothesis that proposed a lower incidence of infections in early childhood as an explanation for the rise in immune-mediated hypersensitivities (88). Although childbirth (natural vs. cesarean section), duration of breastfeeding, and the time of gluten introduction into the child's diet have been discussed, the associations are far from clear, and the cumulative incidence of CD in later childhood was similar independent of breastfeeding or timing of gluten introduction (89, 90). Currently, there is no possibility to prevent CD (91).

#### Symptoms

The clinical appearance of CD is highly variable and can range from asymptomatic to full-blown symptoms due to the multisystemic nature of CD (92). Classical gastrointestinal symptoms are chronic diarrhea, abdominal pain, vomiting, and steatorrhea. Extraintestinal manifestations include chronic fatigue, night blindness, anemia, osteoporosis, thyroid dysfunction, and reproductive disease (93) and are usually caused by generalized malabsorption of essential nutrients, e.g., vitamins and minerals. Slow growth rates and delayed sexual maturation are known complications of CD in children and adolescents. The characteristic feature of CD is damage to the upper small intestine (duodenal bulb, duodenum, proximal jejunum) characterized by increased infiltration of intraepithelial lymphocytes (IELs), crypt hyperplasia, and partial to total villous atrophy. The degree of mucosal damage is classified according to Marsh–Oberhuber (into types 0, 1, 2, 3a–c, and 4) (94) or Corazza (into grades normal, A, B1, and B2) considering the numbers of IELs per 100 enterocytes, ratio of villous height to crypt depth, and degree of villous atrophy [overview in Ludvigsson et al. (7)]. Asymptomatic CD patients have minimal or no symptoms, but they have increased CD-specific serum antibody levels and villous atrophy. Potential CD patients have no symptoms and a normal mucosa, but increased levels of antibodies indicating an increased risk of developing CD. Most individuals with asymptomatic or potential CD have not yet been diagnosed for CD. They may be diagnosed through case-finding approaches in at-risk populations such as first-degree relatives of CD patients or patients with autoimmune diseases (e.g., diabetes mellitus type I, autoimmune hepatitis, autoimmune thyroid disease) or genetic disorders (e.g., Down syndrome) with known associations to CD (95–97).

Refractory CD, classified into type I (normal IEL phenotype) and II (aberrant IEL phenotype), is a rare but very serious complication, which is characterized by persistence of CD-specific symptoms and mucosal lesions despite a permanent, strict gluten-free diet (GFD) (98). Type I can usually be treated with corticosteroids, but type II imposes a serious risk of progression to enteropathy-associated T-cell lymphoma, small intestinal adenocarcinoma, and ulcerative jejunitis.

#### Diagnosis

The diagnosis of CD requires a high level of clinical suspicion and a stepwise approach. The diagnostic scheme may consist of five steps: (a) clinical history and symptoms, (b) serology, (c) small intestinal histology, (d) response to a GFD, and (e) HLA status. The most recent diagnostic algorithm developed for children by the European Society for Pediatric Gastroenterology, Hepatology, and Nutrition (ESPGHAN) recommends testing symptomatic patients or risk groups for IgA anti-tissue transglutaminase antibodies (TGA) and total serum IgA to exclude IgA deficiency. If IgA TGA is negative and total IgA is normal, CD is unlikely. If IgA TGA levels are <10 × the upper limit of normal (ULN), the patient has to undergo upper endoscopy with multiple biopsies including at least four from the descending part of the duodenum and at least one from the duodenal bulb. If IgA TGA levels are >10 × ULN, the patient is additionally tested for IgA anti-endomysium antibodies (EMAs). If EMAs are positive, the diagnosis of CD is established, and a GFD is initiated with subsequent follow-up for improvement of symptoms and decline of antibodies to substantiate the correct diagnosis. If EMA is negative, additional testing including biopsies is needed. Gluten challenge and repetitive biopsies are only necessary in ambiguous cases. Especially in children, biopsies are avoided, if possible, but are still recommended, if there are uncertainties related to the performance of the kit used to measure IgA TGA (99). The same algorithm essentially applies to adults, with the only difference that a duodenal biopsy is advisable in almost all cases because adults are more likely to have an alternative diagnosis or to be non-responsive to a GFD (100). There are many clinical situations that require special considerations and additional tests, such as patients under the age of two and patients with selective IgA deficiency, other immunodeficiencies, or on immunosuppressive medication (101). In case of potential CD, regular follow-up on a normal diet is recommended, whereas patients with suspected, but undocumented CD already adhering to a GFD need to undergo gluten challenge (102). The final confirmation of CD is established by a clinical, serological, and histological response to a strict GFD. Genetic tests based on HLA-DQ2 and -DQ8 alleles can be used to rule out CD in ambiguous cases because of their high negative predictive value. The need for mass screening for CD in the general population is discussed controversially (103). At present, screening is recommended for close relatives of CD patients and for persons with diseases known to be associated with CD such as autoimmune diseases.

#### Pathomechanism

The pathomechanism of CD is complex and involves both adaptive and innate immune responses (104). Due to their high contents of proline and glutamine, gluten proteins are resistant to complete digestion by human gastrointestinal enzymes. As a consequence, peptides with a length of nine amino acid residues and more stay intact and pass the small intestinal epithelium, either by the trans- or the paracellular routes into the lamina propria (105). More than 1,000 CD-active (CD-toxic and/or -immunogenic) gluten peptides derived from gliadins, glutenins, secalins, hordeins, and avenins have been identified, seven of which are classified as CD-toxic (tested *in vivo* or on organ cultures), five of which are classified as CD-toxic and CD-immunogenic (tested by T-cell proliferation assays), and the vast majority of which are classified as CD-immunogenic (106). Typical features of CD-active peptides are high contents of proline (P) residues and a left-handed polyproline II helical conformation that protects from enzymatic degradation as well as high contents of glutamine (Q) residues that serve as substrates for deamidation or transamidation by human tissue transglutaminase (TG2) (107). Having reached the lamina propria, gluten peptides with QXP or QXXJ motifs (X, any amino acid, J, hydrophobic amino acid) are specifically deamidated (Q → E, introduction of a negatively charged glutamic acid residue) or transamidated by TG2 (either to itself or to other lysine donors) (108, 109), whereas QP and QXXP motifs are left unmodified (110). Then, gluten peptides are bound to the heterodimeric HLA-DQ2 or -DQ8 receptors on the surface of APCs. Deamidation at positions 4, 6, and 7 of the gluten peptides strongly favors binding to HLA-DQ2, while HLA-DQ8 prefers deamidation at positions 1 and 9 (respective locations of positively charged amino acid residues in the binding pockets of HLA-DQ2 or -DQ8) (86). APCs subsequently present gluten peptides to the receptor of naive CD4^+^ T cells and promote T-cell activation and differentiation into gluten-specific inflammatory effector T cells. On the one hand, these gluten-specific CD4^+^ T cells secrete proinflammatory cytokines, such as interferon-γ and tumor necrosis factor (TNF)-α, which stimulate the release and activation of matrix metallopeptidases. These break down extracellular matrix proteins and thus lead to the destruction of the small intestinal epithelium (proinflammatory Th1-pathway, adaptive immune response). On the other hand, gluten-specific CD4^+^ T cells help B cells that carry internalized TG2-gluten peptide complexes. This results in B-cell activation and differentiation into plasma cells that produce IgA and IgG antibodies against gliadin and deamidated gluten peptides as external antigens, and against endomysium and TG2 as autoantigens (anti-inflammatory Th2-pathway, adaptive immune response) (84, 111).

Additionally, gluten peptides stimulate the innate immune response and trigger the secretion of interleukin (IL)-15 by activating enterocytes, macrophages, and dendritic cells. As a result, lymphocytes are stimulated to express the receptor NKG2D and epithelial cells to express MICA (major histocompatibility complex class I chain-related molecule A), the ligand for NKG2D. Once MICA has bound to NKG2D, IELs start to destroy epithelial cells. The key factor explaining why tolerance to gluten is lost involving the switch from a tolerogenic Foxp3^+^ regulatory T-cell response to a proinflammatory Th1 response still remains elusive (112).

#### Treatment

A strict GFD with a daily gluten intake below 20 mg is currently the only safe and efficient therapy for CD to fully restore patient health. Dietetic gluten-free products are made from safe cereals (e.g., corn, rice, sorghum, or millet), pseudocereals (e.g., amaranth, buckwheat, or quinoa), other sources of flour or starch (potatoes or chestnut), and “gluten replacers” (xanthan or guar gum) (113). Owing to the restricted availability and high costs of gluten-free alternatives and their poorer quality (114), many CD patients regard the GFD as a substantial burden that decreases quality of life, especially when eating out or traveling. Therefore, their most pronounced desire is the development of a pill or a vaccine that will allow them to eat gluten-containing foods, at least sometimes. Therefore, alternative therapies targeting different steps in the pathomechanism of CD are in various stages of development. Therapies that have already reached clinical trial stage include glucocorticoids (budesonide), oral administration of gluten-degrading enzymes (ALV003, a mixture of cysteine endopeptidase B, isoform 2, and a prolyl endopeptidase from *Sphingomonas capsulata*), oral intake of gluten-sequestering polymeric resins [poly(2-hydroxyethylmethacrylate-co-styrene-4-sulfonic acid, sodium salt)], zonulin antagonists (larazotide acetate), vaccination (Nexvax2), probiotics, and hookworm infection (115). All alternative therapies still need to demonstrate that they are tolerable and safe and have no adverse long-term side-effects. Especially for gluten-degrading or -removing agents, the amount of gluten that can be safely ingested needs to be determined considering all other ingredients of the meal that may hinder the efficacy of the agent. Finally, the benefits and risks of alternative therapies have to be carefully weighed against the GFD to ensure that CD patients receive the best treatment option available.

### Dermatitis Herpetiformis and Gluten Ataxia

With a prevalence of 0.03–0.07%, dermatitis herpetiformis (DH) is often referred to as the skin manifestation of CD. Both diseases are caused by gluten, respond to treatment with a GFD, and share the genetic predisposition caused by HLA-DQ2 or -DQ8. Typical symptoms are intense itching and burning papules, macules, and blisters especially on the elbows, knees, and buttocks. DH is ideally diagnosed by direct immunofluorescence biopsy of unaffected skin close to an active lesion that reveals granular IgA deposits in the papillary dermis. The autoantigen in DH is epidermal transglutaminase (TG3). The most likely pathogenic route starts from potential or asymptomatic CD in the small intestine with secretion of IgA against TG2 and TG3 into the blood circulation and results in the deposition of TG3 and IgA against TG3 in the papillary dermis. Due to the presence of active TG3, IgA–TG3 complexes are formed and crosslinked to fibrinogen in the skin (116).

Gluten ataxia (GA) can be regarded as rare neurological manifestation of CD and is defined as idiopathic sporadic ataxia characterized by the presence of IgA or IgG against gliadin in the blood. GA presents with gait and lower limb ataxia, nystagmus, and other visual disorders. Up to 40% of patients show small intestinal damage as in CD and up to 60% of patients have evidence of cerebellar atrophy. Much like in DH, the autoantigen in GA is the primarily brain-expressed transglutaminase (TG6) and IgA against TG6 are serological markers for GA. TG6 and IgA against TG6 accumulate in the brain stem and cerebellum causing infiltration of white matter with lymphocytes and irreversible loss of Purkinje cells in the cerebellar cortex. Therefore, a fast diagnosis of GA and treatment with a GFD are essential to prevent progression of cerebellar dysfunction (117).

### IgE-Mediated Wheat Allergies

WAs are defined as adverse immune responses to wheat proteins that reproducibly occur in affected individuals within minutes to hours after exposure (118, 119). A wide variety of wheat proteins including gluten and non-gluten proteins have been identified as allergens [overviews in Brouns et al. (120), Juhasz et al. (121), and Tatham and Shewry (122)]. Depending on the route of allergen exposure and the underlying pathomechanism, WAs can be classified into food allergy, wheat-dependent exercise-induced anaphylaxis (WDEIA), respiratory allergy, and skin allergy. The prevalence estimates for WAs depend on the assessment method used, but range from 0.1% (positive food challenge) to 3.6% (lifetime self-reported prevalence) (123).

Common diagnostic procedures include patient history reporting reproducible symptoms after allergen exposure, skin prick tests, analysis of specific IgE antibodies, or functional assays such as *in vitro* basophil activation tests and oral food challenge. While oral food challenge is generally regarded as a gold standard for the diagnosis of wheat allergy and can offer clarity in ambiguous cases, it is difficult to undertake in routine clinical practice and puts patients at risk of experiencing a severe allergic reaction. The treatment for wheat allergy mostly involves avoidance of exposure to allergens, either in the form of flours and flour dust or elimination of wheat products from the diet. Antihistamines or corticosteroids can be used to treat acute cases (124, 125).

The mechanism of IgE-mediated allergies includes two phases. The first step is sensitization to the allergen upon initial contact, followed by an allergic reaction upon reexposure to the allergen (126). When an allergen is encountered for the first time, it is internalized by APCs (e.g., dendritic cells) and presented to naive CD4^+^ T cells. In the presence of cytokines, the naive CD4^+^ T cells become activated and differentiate into Th2 cells that subsequently produce different ILs. IL-4 turns on IgE-producing B cells and sustains the development of Th2 cells, IL-5 activates eosinophils, IL-9 enhances IgE production, mast cell growth and expression of the high-affinity IgE receptor (FcεRI), and IL-13 acts on epithelial cells to stimulate mucus secretion. The secreted IgE antibodies bind to FcεRI on the surface of mast cells and basophils. When the mast cell carrying IgE antibodies is reexposed to the allergen, the multivalent allergen crosslinks two adjacent IgE antibodies and the underlying FcεRI. This bridging leads to mast cell degranulation with discharge of primary (preformed) mediators (histamine, neutral peptidases, acid hydrolases, and proteoglycans such as heparin and chondroitin sulfate) and synthesis and release of secondary mediators (leukotrienes, prostaglandin D2, platelet-activating factor, cytokines, and chemokines). The rapid release of histamine and leukotrienes is responsible for the intense early allergic response characterized by wheezing, sneezing, urticaria, and mucus secretion. The survival of mast cells and enhanced expression of the FcεRI receptor is sustained by signals from these receptors, thus providing a mechanism of amplification. In the following phase, eosinophils are activated by IL-5 and attracted to the site of the immediate reaction by chemokines (e.g., eotaxin). By producing cytokines, leukotrienes, and proteins (major basic protein and eosinophil cationic protein) that are toxic to epithelial cells, the inflammatory response is amplified and sustained without additional exposure to the allergen. These events lead to the late allergic response, which involves further wheezing, nasal blockage, and eczema. Basophils play a similar role to that of mast cells, but they circulate in the blood rather than being present in the affected tissues.

#### Respiratory and Skin Allergy to Wheat

Respiratory WA comprises baker's asthma and allergic rhinitis, which are allergic responses to the inhalation of flours and dust from wheat and other cereals (rye, barley) known since Roman times. Both allergies rank among the most prevalent occupational diseases and affect 1–10% (baker's asthma) and 18–29% (allergic rhinitis) of bakers, millers, and confectioners. Depending on the severity of the reaction, vocational retraining may be necessary. More than 100 IgE-binding proteins were identified, of which chloroform–methanol-soluble (CM-) ATIs, lipid transfer (LTP) and non-specific lipid transfer proteins (nsLTP) are the major allergens (127).

Contact urticaria is an allergic reaction on the skin following contact with an eliciting allergen (128). Typical symptoms of urticaria are localized wheal-and-flare reactions such as hives and a raised, burning, and/or pruritic swelling of the skin, often accompanied by angioedema that appear within 10–30 min after allergen exposure and fade away within hours. In contrast, contact dermatitis is accompanied by the appearance of large, burning, and itchy rashes, blisters, and wheals that take several days to weeks to heal (129). Similar to respiratory WA, millers, bakers, and flour handlers are most frequently affected.

#### Food Allergy to Wheat

Wheat is the third most common cause of food allergy (only surpassed by milk and egg) and has to be labeled on prepacked foods according to Codex Alimentarius Standard 1–1985 (130) and also non-prepacked foods according to EU regulation 1169/2011. Wheat food allergy occurs within a few hours of wheat ingestion and may present with symptoms on the skin (e.g., atopic dermatitis, urticaria, angioedema), in the respiratory tract (e.g., wheezing, bronchial obstruction), in the gastrointestinal tract (e.g., abdominal pain, bloating, diarrhea), and even anaphylaxis. In comparison to other allergies such as peanut allergy, the dose of wheat proteins needed to trigger allergic reactions is usually quite high (about 1 g), but may also be lower (10 mg) depending on individual sensitivities (131). The causative factors are non-gluten proteins as well as gluten proteins, with ATIs 0.19, CM1, CM2, CM3, and CM16, LTP, and nsLTP as well as α-gliadins, γ-gliadins, and HMW-GS as major allergens (132). The IgE-binding epitopes derived from the repetitive sequences of gluten proteins such as QFPQQQFPQQ (ω5-gliadins), QQSFPLQPQQ (ω1,2-gliadins), VQQQQFPGQQ (α-gliadins), QQLPQPQQP (γ-gliadins), and SQQQPPF (LMW-GS) with the consensus motif QQX_1_PX_2_QQ (with X_1_ being L, F, S, or I and X_2_ being Q, E, or G) are different to those reported for CD (133).

#### Wheat-Dependent Exercise-Induced Anaphylaxis

WDEIA is a special form of WA because wheat intake alone does not trigger the allergic reaction, but in combination with augmenting cofactors, such as physical exercise. Further cofactors are alcohol, acetylsalicylic acid (aspirin®), and other non-steroidal anti-inflammatory drugs and stress (134, 135). The estimated prevalence of WDEIA is <0.1%. Clinical features range from urticaria and angioedema to dyspnea, hypotension, collapse, and anaphylactic shock. The major triggers are ω5-gliadins and HMW-GS, but other gluten protein types may also be involved. A special form of WDEIA is an allergic reaction caused by epicutaneous sensitization with hydrolyzed wheat proteins in cosmetics (136). Although WDEIA is regarded as the best-studied model of cofactor-induced anaphylaxis, it is still not clear how exactly the cofactors act as adjuvants causing the reaction. A decreased activation threshold of mast cells and basophils has been discussed, but it appears to be more likely that cofactors increase the bioavailability of allergens by promoting small intestinal permeability (137).

### Non-IgE-Mediated Wheat Allergies

Non-IgE-mediated food allergies are well-known in children under the age of three, and they are divided into three main clinical conditions: food protein-induced (FPI) enterocolitis syndrome, FPI proctocolitis, and FPI enteropathies. The pathomechanism is currently not well-known, but these conditions are characterized by high levels of IL-13 and TNF-α as drivers of intestinal epithelial damage and eosinophil infiltration. The most common trigger for all three conditions is cow's milk, but soy, rice, and wheat have also been reported (138). Non-IgE-mediated food allergies are less well-recognized in adults (139), but there is an increasing body of evidence that they may be under-recognized. NCGS might in fact be a non-IgE-mediated food allergy because of patient history (food allergy during childhood or presence of atopic diseases) and serological and histological findings such as positive serum anti-gliadin antibodies, *in vitro* basophil activation, and presence of eosinophils in the intestinal mucosa (140).

### Non-Celiac Gluten Sensitivity

NCGS, frequently termed gluten or (non-celiac) wheat sensitivity, may be defined as a gluten (wheat)-dependent disorder with symptoms similar to CD, but usually normal small intestinal mucosa (141). Moreover, NCGS is characterized by the lack of serum TG2 antibodies and the missing association to HLA-DQ2/8 alleles. The exact prevalence is still unknown and may be similar to that of CD with reports ranging from about 1% in El Salvador (13) and Mexico (11) to 1.7% in Brazil (10), but may also be higher with up to 6% (142). The clinical differentiation of NCGS from other WRDs is difficult due to several common features: similar symptoms, wheat proteins as the triggering factor, and a GFD as recommended treatment (78). Typical gastrointestinal manifestations of NCGS are abdominal pain, bloating, and chronic diarrhea. Frequent extraintestinal complaints include headache, “foggy mind,” fatigue, anxiety, depression, numbness in the legs, arms and fingers, and joint pain (143). In contrast to CD, NCGS is not associated with malabsorption, nutritional deficiencies, or increased risk for autoimmune diseases or malignancy.

Because symptoms disappear on a GFD, gluten proteins have been considered as a precipitating factor (144), but it is still not clear whether gluten is responsible. Other wheat constituents such as non-gluten proteins and FODMAPs might be additionally responsible for NCGS (145). Recent studies proposed that ATIs contribute to the development of NCGS by an innate immune response mediated by the toll-like receptor pathway (146). Due to the absence of NCGS-specific biomarkers, the diagnosis is currently made by exclusion of CD, WA, other food intolerances, and irritable bowel syndrome (147). The diagnosis is definitely proven by oral wheat challenge after at least 3 weeks on a GFD and the subsequent occurrence of typical symptoms (148). A GFD is recommended as treatment, whereby symptoms usually improve rapidly. In contrast to CD, where strict GFD has to be maintained, patients with NCGS could adapt a more liberal diet reducing the gluten intake by ≈90% (146).

### Irritable Bowel Syndrome

Irritable bowel syndrome (IBS) is the most common and extensively evaluated functional bowel disorder (149). Its prevalence in adult individuals has been estimated in a range from 5 to 20%. IBS is not a single disease but rather a symptom cluster resulting from diverse pathologies (150). Typical symptoms, including abdominal discomfort and pain, gas, bloating, and diarrhea with and without constipation, are similar to those of CD, NCGS, intestinal bacterial overgrowth, and lactose intolerance (151). The Rome IV criteria categorize IBS by the most predominant presenting symptoms: diarrhea, constipation, mixed, or unspecified (152). The majority of patients perceive their symptoms as being related to specific meals, in particular, foods rich in carbohydrates. Wheat is regarded as one of the most relevant IBS triggers, although which component of this cereal is involved remains unclear (153). Gluten, FODMAPs, other wheat proteins, for example, ATIs, have been suggested as possible factors for symptom generation. The experimental and clinical evidence on the role of gluten/wheat in IBS has been presented by Volta et al. (154). Some types of IBS, especially diarrhea-predominant cases, show symptomatic improvement on a GFD (155). Today, the diagnosis is not only based on the exclusion of other food hypersensitivities, but on positive diagnosis using symptom-related criteria (“Rome III diagnostic criteria”) (156). The pathomechanism of IBS is not completely understood; factors important to the development of IBS include alterations in the gut microbiome, intestinal permeability, gut immune function, motility, visceral sensation, brain–gut interactions, and psychosocial status (157). Strategies for treatment are based on general recommendations such as avoidance of foods rich in fat and carbohydrates, dairy products, caffeine, and alcohol, on the one hand, and increased intake of DF, on the other hand. If patients can associate the ingestion of certain foods with the complaints, an improvement may be achieved after restriction of these foods. Diarrhea-predominant patients are advised to test a GFD for several months (155).

## The Sources of Confusion Around the Sad Face of Wheat

### Unsubstantiated Statements Blaming Wheat

Over the last decade, wheat has been the center of a vigorous debate related to health and nutrition, and it has gained an increasingly negative reputation among the Western population. The first and main source of confusion arose from several pseudoscientific books such as “Wheat Belly” (158) and “Grain Brain” (159), numerous media reports and statements by celebrities promoting the overall impression that wheat consumption has adverse health effects for the general population. The main statements claimed that wheat consumption was the cause of overweight and obesity, that wheat caused a whole number of other disorders such as diabetes type 2, asthma, reflux disease, sleep disorders, neuronal complaints, etc., that wheat bread had an overly high glycemic index, that wheat contained opioids that caused addiction, and that modern wheat had been genetically modified and contained unique toxic proteins that caused the higher prevalence of WRDs. In conclusion, the general population was advised to avoid the consumption of wheat products.

However, these books intermingle sound scientific evidence with theoretical, controversial, and wrong statements and make it virtually impossible for consumers to separate the truth from myths. The essential summary that “foods made from wheat make people sick, stupid, fat, and addictive” (158, 159) led to great uncertainties among consumers. As a consequence, the popularity of a GFD has increased, with up to 5% of the population in New Zealand reporting gluten avoidance (160) and up to 13% of the UK population self-reporting a WRD (9). The consumption of gluten-free foods has significantly increased over the last years, up from a global retail sales value of 1.95 × 10^9^ USD in 2012 to 3.84 × 10^9^ USD in 2017, with further increases projected in the coming years (161). The reasons why some people voluntarily adopt a GFD include that they think it might help them reduce weight, that they perceive a GFD as healthier and better for their overall well-being and that they self-diagnosed a WRD.

To counteract the increasing uncertainty among consumers, numerous counterstatements of the scientific community [e.g., (120, 162–165)] have emphasized that wheat consumption is safe for the vast majority of the population and that wheat avoidance is only necessary after medical diagnosis of a true WRD. These reviews have compiled convincing evidence to refute the abovementioned statements and assert that the regular consumption of whole-grain products is associated with reduced risks of type 2 diabetes and of colorectal cancer, likely reduced risks of colon cancer and cardiovascular diseases, and more favorable weight management (166).

### The Increasing Prevalence of Wheat-Related Disorders

The second source of confusion related to wheat consumption is based on the increasing amount of evidence from well-founded epidemiological studies that show a rise in the prevalence of WRDs over the past 50 years. While this rise can be partially explained by better diagnostics and improved awareness, recent reports, e.g., from Denmark (167), Italy (168), the United States (169), and worldwide (79) show that the prevalence of CD has indeed increased over time. The same is reported for NCGS (170) and also for allergies and autoimmune diseases, in general (171). Despite ongoing research, the underlying causative factors have not been unambiguously identified so far. WRDs are initiated through a loss of immunotolerance to wheat proteins at a certain point in time, but the factors causing this initial loss are the subject of ongoing investigations and may also be different depending on the genetic predisposition, dietary habits, and overall lifestyle of each individual.

The most likely factors include the hygiene hypothesis that was originally based on the observation that the decreased frequency of overall infectious and parasitic diseases was inversely correlated to the increased frequency of allergic and autoimmune diseases seen in industrialized countries since the 1950s (88). The hygiene hypothesis is supported by studies on migrants, who are as likely to develop an autoimmune disease as individuals in the host country with a high incidence of autoimmune diseases, even if they originally came from a country with a low incidence of autoimmune diseases, but moved at a young age (172). Studies in mice support the protective effects of pathogenic bacteria, viruses, and parasites on autoimmunity and the additional positive effects of commensals that stimulate innate and adaptive immune regulatory pathways (88). In contrast, a lack of physical activity, a lot of time spent indoors, and a diet rich in saturated fats and digestible carbohydrates, but poor in DF, are associated with a loss of microbial diversity of the gut, skin, and other tissues and a subsequent loss of symbiotic relationships with parasites and bacteria that used to exist during human evolution (173–175). Additional factors contributing to changes in the microbiome are antibiotics and vaccinations and decreased exposure to airborne bacteria, all of which may contribute to alterations in intestinal permeability. Intestinal barrier dysfunction has been associated with a variety of intestinal and systemic diseases, including CD (176).

Seen from the side of wheat and related cereals, changes in protein composition due to breeding, heat and cold stress, or agricultural practices have been postulated as potential contributors to a higher immunostimulatory potential of modern wheat species compared to landraces and heritage wheats. While protein expression patterns do differ between wheat species, i.e., diploid einkorn, tetraploid emmer, and durum wheat as well as hexaploid spelt and common wheat (177–179), there are currently too few comparative *in vitro* or *in vivo* studies available to allow a precise assessment as to whether these differences might be related to the prevalence of WRDs or not. For example, the 33-mer peptide from α2-gliadin that is frequently described as the immunodominant peptide in CD, was only detected in common wheat and spelt, but it was not present in emmer, durum wheat, or einkorn (180). Ancient wheats like einkorn, emmer and spelt were suggested to provide health benefits compared to common wheat, but recent reviews collected evidence demonstrating that they differ little in their composition. Thus, ancient wheats do not appear to be “healthier” than modern wheats (41), with some exceptions, e.g., high lutein and steryl ferulate contents in einkorn (181).

**Figure 3:**
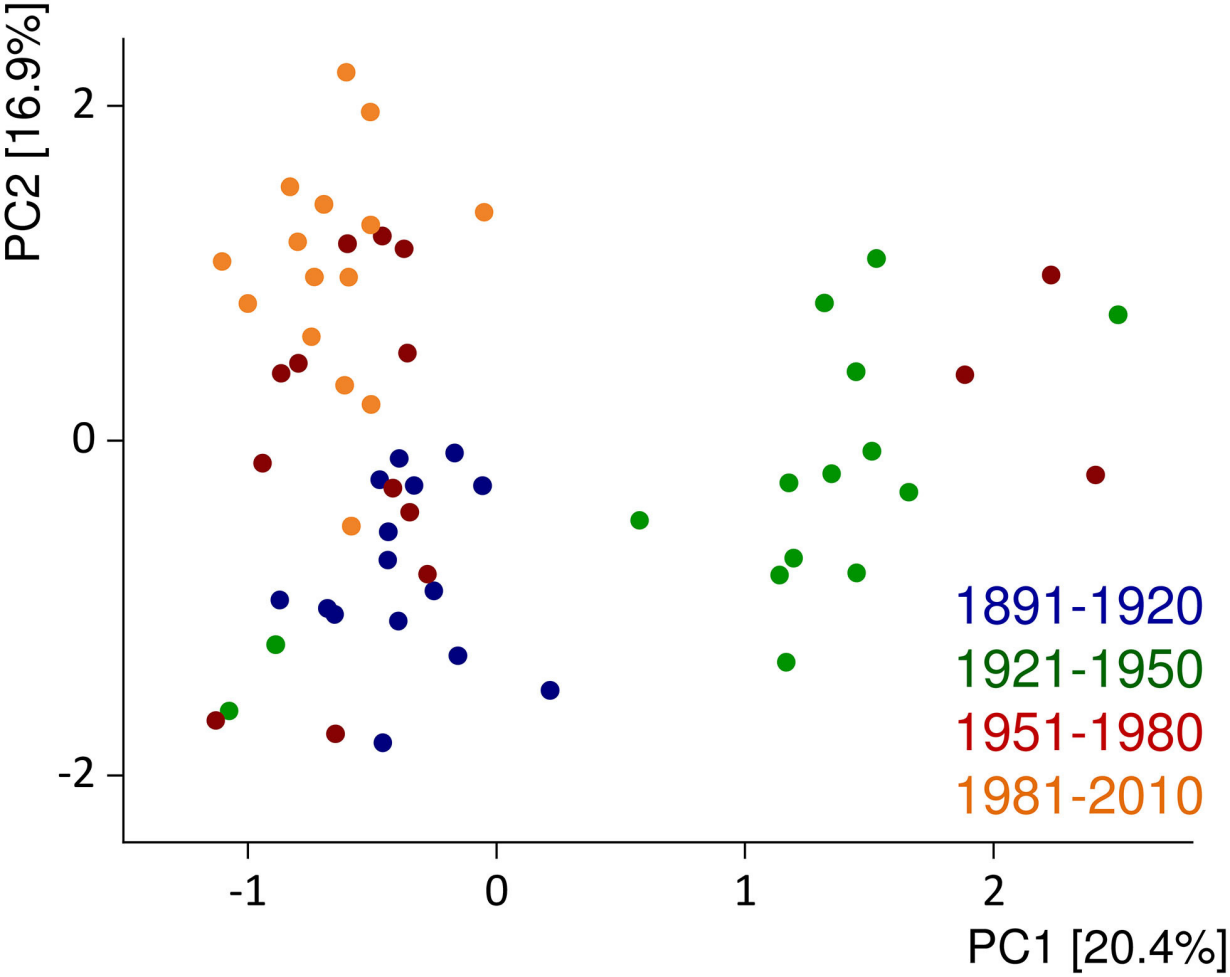
Principal component (PC) analysis biplot of protein fingerprints of albumins/globulins, gliadins, and glutenins relative to the sum of extractable proteins. The data are displayed for 60 German winter wheat cultivars first registered from 1891 to 2010 and show the average of three harvest years (2015–2017). Figure modified from Pronin et al. (191).

Several comparative studies on old and modern cultivars within the species *Triticum aestivum* set out to study the influence of breeding during the last century (182). So far, the results are somewhat inconclusive because one study from Canada reported an increase (183), two studies from the United States essentially reported no change (184, 185), and three others from the United Kingdom, Germany, and the United States reported a decrease in protein contents over time (186–188). All studies report a substantial influence of the growing conditions on the content and composition of wheat proteins. Regarding protein composition, an increase in glutenins and a decrease in gliadins and gliadin/glutenin ratios, but essentially no changes for albumins/globulins and gluten, were observed in German winter wheat cultivars from 1891 to 2010, all grown at the same location in three consecutive years (189). Similar results were reported by Ozuna and Barro (190). Principal component analysis of the chromatographic fingerprints of albumins/globulins, gliadins, and glutenins showed a cluster formation of the most modern cultivars (first registered from 1981 to 2010) and of the oldest ones (1891–1920), but with exceptions and samples from 1951 to 1980 in between. The largest variability in protein profiles was observed for the samples from 1921 to 1950 [Figure 3, (191)]. Altogether, the evidence, so far, points to the conclusion that old and modern wheat cultivars do show changes in protein composition due to breeding, but so far, none of these changes seems to be linked to the prevalence of WRDs.

As wheat is part of a huge variety of products, the amounts of immunoreactive proteins in the end-product that is eventually consumed is more important than in the original flour. Wheat processing techniques as well as strategies to reduce exposure have been extensively reviewed recently (165). The use of ungerminated grains, of refined white flour instead of wholegrain flour, of fast straight-dough yeast fermentation instead of diverse and long sourdough fermentations, as well as the use of wheat gluten as a technofunctional additive in a number of food products (192) have been discussed as additional factors that may contribute to causing WRDs (165, 193). Consumption of these wheat products may have increased the total amount of gluten in the diet, thus surpassing a certain threshold level necessary to trigger WRDs. However, credible data about adverse effects of modern wheat processing are not available, and no epidemiological studies have evaluated the contribution of modern processing on the increasing prevalence of WRDs. For example, there is no proof that modern bread making, including short and non-acidic fermentation of doughs or addition of vital gluten, resulted in higher gluten immunoreactivity. Epidemiological data demonstrate that countries, in which long-fermented sourdough breads are common (e.g., Finland and Sweden) even have higher CD prevalences than countries, where short-fermented yeast-leavened breads are consumed almost exclusively (e.g., Italy and Spain) (194).

### The Unknowns

The third source of confusion mostly arises from the fact that the triggers for NCGS and wheat-sensitive IBS have not been clearly identified, so far (140), partly due to short-comings in the design of the nutritional intervention studies, placebo and nocebo effects (195), and/or insufficient characterization of the wheat product or wheat extract administered. The constituents that are discussed as causes of NCGS are gluten, non-gluten proteins (e.g., ATIs), and FODMAPs. In many cases, products containing wheat flour were used for oral food challenge, and in this case, gluten, non-gluten proteins, and FODMAPs were present (196). In other cases, wheat gluten was used, but these isolates also contain non-gluten proteins, so that the effects of gluten and ATIs cannot be clearly distinguished (195, 197). Gluten, as a cause of NCGS, has mostly been inferred from the fact that NCGS patients' symptoms are alleviated if they follow a GFD, but several studies have reported that gluten may not be the causative factor in NCGS, but rather FODMAPs (195, 198). ATIs were identified as triggers of innate immunity via the toll-like receptor 4, and they have been implicated in causing NCGS (146) and acting as adjuvants of other inflammatory diseases (78).

Furthermore, there is a significant overlap of symptoms of CD, NCGS, and IBS. It is clear that CD patients need to follow a strict GFD, and no positive effects of a GFD or a low FODMAP diet have been proven for healthy individuals (199). In between, both NCGS and IBS patients benefit from a low FODMAP diet, but even more so of a GFD, probably because of a multifactorial etiology of NCGS that combine a function effect caused by FODMAPs with a mild immune reaction combined by a dysbalance of microbiota (200). In most cases, gluten-free raw materials are naturally low in FODMAPs and also low in ATIs, so that a GFD is low in all potential causes for NCGS. In this context, sourdough fermented breads may also be better tolerated by NCGS and IBS patients, because *Lactobacillaceae* and *Bifidobacteriaceae*, as well as fungi and yeasts, possess enzymes capable of degrading gluten and FODMAPs (201). Due to the overlap between a low FODMAP diet and a GFD, it is likely that patients with NCGS, especially self-diagnosed ones, are more likely to suffer from IBS. *Vice versa*, there is a subgroup of NCGS patients among the IBS patients (140). A FODMAP-restricted diet is recommended to treat IBS, but not in the long term, because FODMAPs are also part of DF, and a complete elimination will have a negative effect on gut microbial diversity. Further well-designed dietary intervention studies with appropriate controls and sufficient characterization of the challenge materials are still needed to identify the causes of NCGS and also differentiate which patients will benefit from a GFD or rather a low FODMAP diet.

## The Smiling Face of Wheat Prevails

In the last 10 years, wheat has received much negative attention because several pseudoscientific books and numerous media reports fueled the overall assumption that wheat consumption makes people sick. Despite the common consumer perception of a GFD as being healthy, gluten-free foods had higher contents of fat, saturated fat, sugar, and salt compared to gluten-containing foods (202). The most common deficiencies on a GFD are insufficient amounts of DF, vitamins, calcium, iron, magnesium, and zinc. Moreover, a strict GFD decisively reduces the quality of life, as CD patients can confirm, and may lead to low compliance (203).

Wheat is among the oldest and most extensively grown crops and one foundation to ensure food security for the increasing world population. Technological advances in breeding, farming, and processing have paved the way for wheat to become one of the most widespread and cheapest raw materials for food and non-food applications. No other food crop supplies humans with such a huge diversity of products from bread to other baked goods and pasta products that serve as staple foods all over the world. Wheat-based foods provide valuable nutrients such as proteins, DF, vitamins, minerals, and bioactive phytochemicals and supply up to 20% of the energy intake of the global population. Additionally, wheat is important for many non-food applications, and a number of wheat constituents like starch and gluten are present in items of daily use. Considering all available evidence, so far, there is no reason to eliminate wheat from the diet, except for individuals suffering from WRDs.

## Author Contributions

HW wrote the original draft. PK and KS wrote, reviewed, and edited the manuscript. All authors contributed to the article and approved the submitted version.

## Conflict of Interest

The authors declare that the research was conducted in the absence of any commercial or financial relationships that could be construed as a potential conflict of interest.
